# Attenuates reactive oxygen species: induced pyroptosis via activation of the Nrf2/HO-1 signal pathway in models of trigeminal neuralgia

**DOI:** 10.1038/s41598-023-44013-w

**Published:** 2023-10-23

**Authors:** Mingxing Liu, Yongyi Wang, Shengli Li, Xiaoqun Hou, Tong Li, Zhiming Xu, Feng Chen, Yong Zhou, Lei Xia, Weimin Wang

**Affiliations:** 1 Department of Neurosurgery, Qingdao Hospital, University of Health and Rehabilitation Sciences (Qingdao Municipal Hospital), No.1 Jiaozhou Road, Qingdao, Shandong Province 266011 People’s Republic of China; 2 Department of Neurosurgery, XinHua Hospital, Shanghai JiaoTong University School of Medicine, Shanghai, 200092 China

**Keywords:** Neuroscience, Myelin biology and repair

## Abstract

In this study, we examined the impact of demyelinating and neuroinflammation on trigeminal neuralgia (TN) by utilizing models of chronic constriction injury to the infraorbital nerve (CCI). The CCI rats were treated with either VX-765 (an inhibitor of caspase-1) or a control solution of PBS/DMSO to observe the effects on neurobehavioral and neuropathological outcomes. The histochemical changes, pyroptosis-related proteins were assessed using immunohistochemistry, Elisa, and western blotting. RSC96 cells were pretreated with belnacasan (VX-765, an inhibitor of caspase-1), Gasdermin D(GSDMD)-targeting siRNAs, cobalt protoporphyrin (CoPP) or zinc protoporphyrin (Znpp) before being exposed to H_2_O_2_. Following these treatments, the Reactive oxygen species (ROS) level, cell viability, percentage of pyroptosis, pyroptosis-related proteins, nuclear factor erythroid 2-related factor 2 (Nrf2) and HO-1 level was measured. The scanning electron microscopy revealed increased ball-like bulge and membrane pore formation in the CCI group. In the CCI and CCI+ Vehicle groups, we found ROS level and expression of pyroptosis-related proteins increased. While, treatment with VX-765resulted in a decreased expression of GSDMD, IL-1, IL-18, and caspase-1 decreased. In the in-vitro study, RSC96 cells showed mild pyroptosis and overall mild edema after being exposed to H_2_O_2_. The ROS level, percentage of pyroptosis, pyroptosis-related proteins, Nrf2 and HO-1 level increased significantly in the H_2_O_2_ group. While, the percentage of pyroptosis and pyroptosis-related proteins decreased significantly in the H_2_O_2_ + VX-765 group, H_2_O_2_ + siRNA group, and H_2_O_2_ + VX-765 + siRNA group. After treatment with HO-1-inhibitor Znpp and HO-1-activator Copp, the percentage of pyroptosis and pyroptosis-related proteins increased and decreased significantly respectively. In conclusions, the pyroptosis of Schwann cell in the CCI model generated the demyelination of TN nerve. The ROS is an upstream event of NLRP3 inflammasome activation which induced eventual pyroptosis. The Nrf2/HO-1 signaling pathway could protect the H_2_O_2_-induced pyroptosis in RSC96 cells.

## Introduction

Trigeminal neuralgia (TN), a common hyperactive cranial nerve disorder, is typically caused by artery compression. The TN was most common in the elderly population, with a morbidity rate of about 4–5 per 100,000^1^. Demyelination in the compressed cranial nerve root has been identified as a crucial component of the TN mechanism^2^. Previous research found that the incidence of TN is higher in multiple sclerosis (MS) patients than in the general population, indicating a link between TN and demyelination^3,4^. Hilton and colleagues were the first to report the presence of trigeminal nerve demyelination in TN patients^5^. Burchiel and colleagues discovered that trigeminal nerve roots in cat and Macaca mulatta monkey models generated extra action potentials in response to stimulation from iatrogenic demyelination^6^. While, the underlining mechanisms of demyelination is not clear.

Pyroptosis, a form of programmed cell death, has indeed been linked to the release of proinflammatory cytokines which could lead to demyelination^7,8^. The NLRP3, a key player in pyroptosis, can activate caspase-1 and subsequently result in the release of proinflammatory cytokines such as interleukin-1 (IL-1) and interleukin-18 (IL-18). McKenzie et al. discovered that multiple sclerosis (MS), which is characterized by demyelination in the central nervous system, is linked to inflammasome activation and pyroptosis^8^. Pyroptosis and inflammasome activation have been linked to the pathogenesis of other CNS diseases, including Alzheimer’s disease, and traumatic brain injury^9–11^. While, there was no study focusing on the role of pyroptosis in the demyelination of TN. Reactive oxygen species (ROS) have been identified as a precursor event to the activation of the NLRP3 inflammasome, contributing to the process of pyroptosis^12^. Factors such as oxidative stress, infection, inflammation, and ischemia can all induce the expression of HO-1, a cytoprotective enzyme that helps to mitigate oxidative stress. The Nrf2/HO-1 signaling pathway has been shown to mediate cellular responses to oxidative stress. Activation of Nrf2 results in the upregulation of HO-1, providing a protective mechanism against oxidative damage. Based on this, it has been suggested that the Nrf2/HO-1 pathway could play a role in pyroptosis, possibly by modulating the production of ROS and subsequent activation of the NLRP3 inflammasome^13,14^. However, more research is needed to fully define the involvement of the Nrf2/HO-1 pathway in pyroptosis and to understand how this might be harnessed for therapeutic benefit in diseases associated with pyroptosis. The potential involvement of this pathway in the pathogenesis of diseases such as TN, where pyroptosis is implicated, is also an exciting area of future investigation.

This study specifically focused on how demyelinating and neuroinflammation, key pathological features in TN, are affected by pyroptosis. We also investigated how Nrf2/HO-1 affected the pyroptosis signaling pathway and the molecular alterations involving the NLRP3 inflammasome pathway. New information on the effects of pyroptosis on demyelinating in TN model is provided by this study.

## Materials and methods

### Reagents, antibodies and plasmids

From Sigma (St. Louis, MO, USA), we bought the propidium iodide (PI, P4170) and Hoechst 33342 (B2261). Belnacasan (VX-765, T6090) was purchased from Topscience Co., Ltd. (Shanghai, China). A kit for detecting apoptosis with annexin V-FITC and PI was bought from Vazyme Biotech Co., Ltd. (Nanjing, China). In the current study, we used the following primary antibodies: IL-1β (ab9722), NLRP3 (ab263899), Caspase-1 (ab207802), IL-18 (ab243091) and GSDMD (ab219800) from Abcam Plc. (Cambridge, UK). We bought the Human IL-1β ELISA Kit (CHE0001) and human IL-18 ELISA Kit (CHE0007) from 4A Biotech Co., Ltd., (Beijing, China). ZnPP (purity ≥ 92%) were purchased from Sigma (SigmaAldrich, USA); H_2_O_2_ and cobalt protoporphyrin (CoPP) were purchased from J&K Scientific (Beijing, China). β-actin (M177-3) was purchased from MBL International (Woburn, MA, USA);

### Animals

In our preliminary experiment, the CCI models using the male rats were more stable and had higher success rate. Consequently, in the study, the adult male Sprague–Dawley rats weighing 180–220 g were used and the study were approved by Qingdao University's Institutional Animal Care and Use Committee. In standard laboratory conditions, free access to water and food were given to the animals. Attempts were made to reduce the number of animals and their discomfort.

### Surgery and drug administration

The trigeminal neuropathic pain model was established using chronic constriction injury of the infraorbital nerve (CCI), following the procedures outlined in previous research. Except for the actual nerve ligation, the animals in the sham groups underwent the same surgical procedure. According to previous research, the successful trigeminal neuropathic pain model had a pain threshold less than 2 g^15,16^.

CCI animals were administered VX-765 via intraperitoneal injection to assess its effects on neurobehavioral and neuropathological outcomes. VX-765 (50 mg/kg) injections diluted in PBS/DMSO were given from the onset of clinical signs (day 14) until the end of the experiment (n = 10). For the control group (n = 10), animals were given injections of the vehicle solution, PBS/DMSO. Mechanical threshold, dynamic allodynia, and pinprick hyperalgesia were all observed after surgery (day 1, 3, 7, 14, 21) in CCI models with or without VX-765 administration^16^.

The investigator blinded to the treatment conditions performed the behavioral experiment. With 20 von Frey filaments (North Coast, USA), the mechanical pain threshold was detected. The 5/0 brush was used to stroke the center of the whisker pad gently to test dynamic allodynia. The response of dynamic allodynia and pinprick hyperalgesia were scored as 0–3 as previous study showed. The allodynia and hyperalgesia scores were reported as the average scores across three trials per rats.

### Specimens and test

With pentobarbital anesthesia (50 mg/kg, intraperitoneally), the animals were perfused with saline followed by 4% paraformaldehyde in 0.1 M phosphate buffer (4 °C, pH 7.2–7.4) through the ascending aorta. Subsequently, the prior incision was reopened, and a 3 mm long infraorbital nerve was quickly removed around the construction sites.

The trigeminal nerve demyelination was studied using electron microscopy. Sections were collected on polyvinyl Formvar–coated grids and examined using electron microscopy (H-600, Hitachi, Japan). G-ratios (axon diameter/nerve diameter) reflect the degree of myelination. Random sampling was performed, and G-ratios were calculated at 20,000× magnification.

The histochemical changes of the trigeminal nerve were detected using HE staining, LFB staining, and Bielschowsky’s method. Severity of inflammation was determined in 5 sections from each animal in a blinded manner. Level of inflammation were scored as 0–4. The scoring for demyelination in LFB-stained sections were scored as 1–3. The axonal damage and loss were scored as0-3. GSDMD, caspase-1, NLRP3, IL-18, and IL-1 were detected using immunohistochemistry, Elisa, and western blotting. We measured the percentage of positive area using LEICA Qwin V3 digital image processing system (Germany). In order to identify positively stained structure, we set the density threshold above background level firstly. An average percentage of area of IR relative to the total area of the coverslips was calculated. A ROS Assay Kit (Nanjing Jiancheng Bioengineering Institute, Nanjing, China) was used to measure the levels of ROS. ROS levels were measured using flow cytometry.

### Cell culture and drug treatment

RSC96 cells were pre-treated for 24 h with VX-765 (50 M, catalog no. inh-vx765-1; InvivoGen), a mixture of three commercially available GSDMD-targeting Dicer-Substrate siRNAs (30 nM), or solvent control. In separate treatment groups, cells were pre-treated with CoPP (40 mM) or ZnPP (10 M) for 24 h before being incubated with H_2_O_2_ (300 µM) for another 24 h. Supernatants were collected and kept at − 80 °C.

Following the various treatments described above, cell viability was assessed using a Cell Counting Kit-8 (CCK-8, Topscience Co., LTD, Shanghai, China). An average was calculated from three independent experiments. Cell viability was calculated as [(Treated: A450–A650)−(Blank: A450–A650)]/[(control: A450–A650)−(Blank: A450–A650)] × 100%.

The fluorescence microscopy (Zeiss LSM710; Carl Zeiss, Oberkochen, Germany) was used to assess cell death after staining with Hoechst 33342 (B-2261, Sigma, St. Louis, MO, USA) and propidium iodide (PI, P4170, Sigma, St. Louis, MO, USA). Cell death was quantitated as the percentage of PI-positive cells relative to the total cell number (Hoechst 33342-positive cells). All experiments were performed at least three times. The annexin V-FITC/PI Detection Kit from BD Biosciences (San Jose, CA, United States) was used to measure pyroptosis. The stained cells were analyzed on the FACSVerse flow cytometer (BD Biosciences, San Jose, CA, USA). Data acquisition and analysis were performed using the Flowjo software (BD Biosciences, San Jose, CA, USA). ROS levels were measured using flow cytometry. The RSC96 cells were gold sputtered and examined with a Hitachi S-4800 scanning electron microscope (SEM) set to 15 kV. GSDMD, caspase-1, NLRP3, IL-18, IL-1, Nrf2 and HO-1 expression in RSC96 cells was detected using immunohistochemistry, immunofluorescence, Elisa, and western blotting. All experiments were performed at least three times.

### siRNA knockdown of GSDMD

Cells were transfected using PrecisionFectin Transfection Reagent and either a combination of three commercially available GSDMD-targeting Dicer-Substrate siRNAs (30 nM) or 30 nM of nontargeting siRNA (TriFECTa RNAi Kit; Integrated DNA Technologies) (BioIntersect). After transfected using siRNAs with 48 h, Cells were exposed to H_2_O_2_ or a solvent control for 24 h (with or without VX-765).

**Figure 1:**
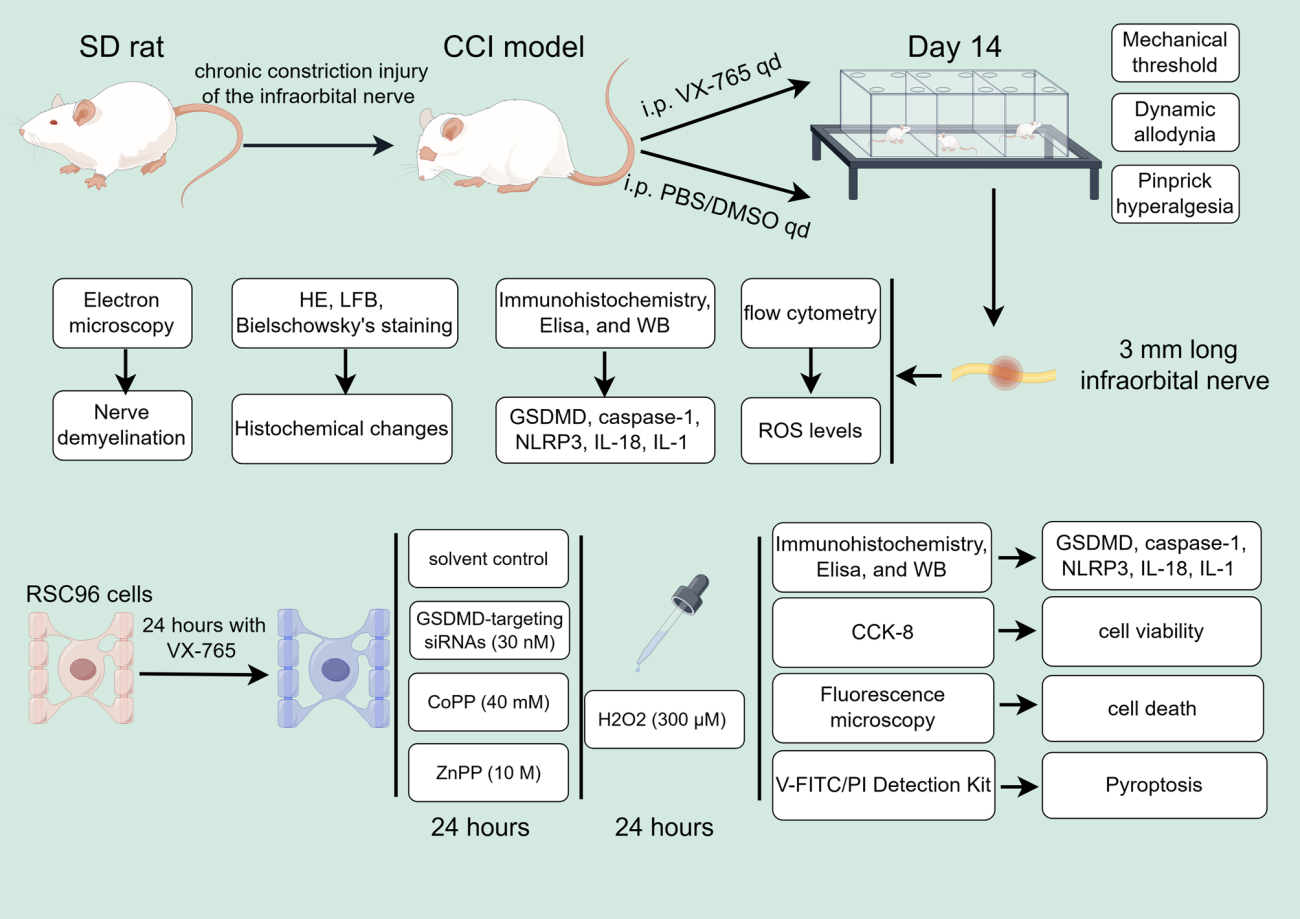
The sketch of the experimental procedure.

I confirm that all methods were performed in accordance with the relevant guidelines and regulations. The above methods were described in detail in the supplementary methods. In order to express the various tasks, measures and a timeline of measurements precisely, we made a sketch of the experimental procedure (Fig. 1).

### Quantification and statistics

The results of the behavioral and immunofluorescence tests are shown as Mean ± SD. With SAS 8.0 Software, differences between groups were statistically analyzed using the two-way ANOVA depending on various variables. We used the Shapiro–Wilk method to test of normality of data. A p value of 0.05 or lower was deemed statistically significant.

### Ethics approval and consent to participate

All the animals were treated in strict accordance with Animal Research: Reporting of In Vivo Experiments (ARRIVE) guidelines, and this study was formally reviewed and approved by the approved by Qingdao University’s Institutional Animal Care and Use Committee.

## Results

### Demyelination of TN nerve and neurobehavioral changes of CCI model

**Figure 2:**
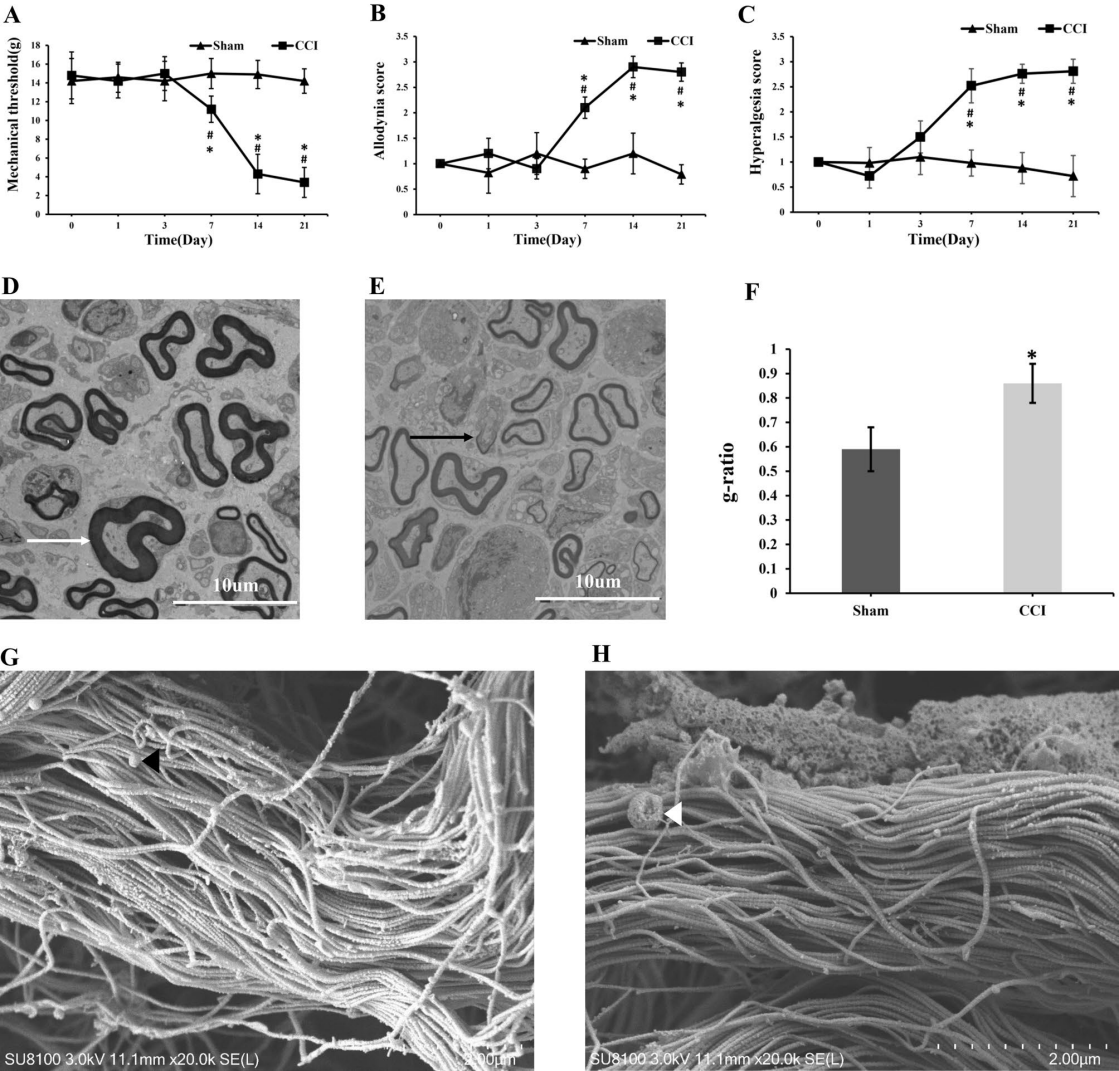
Demyelination of TN nerve and neurobehavioral changes of CCI model. The changes of mechanical threshold, dynamic allodynia and pinprick hyperalgesia scores showed in (**A–C**). *p < 0.05 vs CCI group; # p < 0.05 vs base line. The results of electron microscope analysis of trigeminal nerve are shown in (**D, E**). Scale bar: 10 µm. The CCI group exhibited a higher degree of demyelination (G-ratio) than the control group (**F**). *p < 0.05 vs sham group. The scanning electron microscopy revealed increased ball-like bulge and membrane pore formation in the CCI group (**G**, **H**). Scale bar: 2 µm.

The mechanical threshold significantly decreased after the contraction injury of trigeminal nerve on day 7, reaching its lowest point on day 14 and remaining at that level until the final time point on day 21 (Fig. 2A). Interestingly, the scores for dynamic allodynia and pinprick hyperalgesia showed a significant increase on day 7, reaching their peak on day 14, and staying high until the final time point on day 21 (Fig. 2B, C). Under an electron microscope, we observed homogeneously demyelinated changes in these successful animal models (Fig. 2E), while mild epineurial lesions were observed in the unsuccessful model animals (Fig. 2D). The CCI group showed a higher degree of demyelination (G-ratio) than the control group (Fig. 2F). Additionally, scanning electron microscopy revealed an increased ball-like bulge and membrane pore formation in the CCI group (Fig. 2G, H).

### Histopathological findings

**Figure 3:**
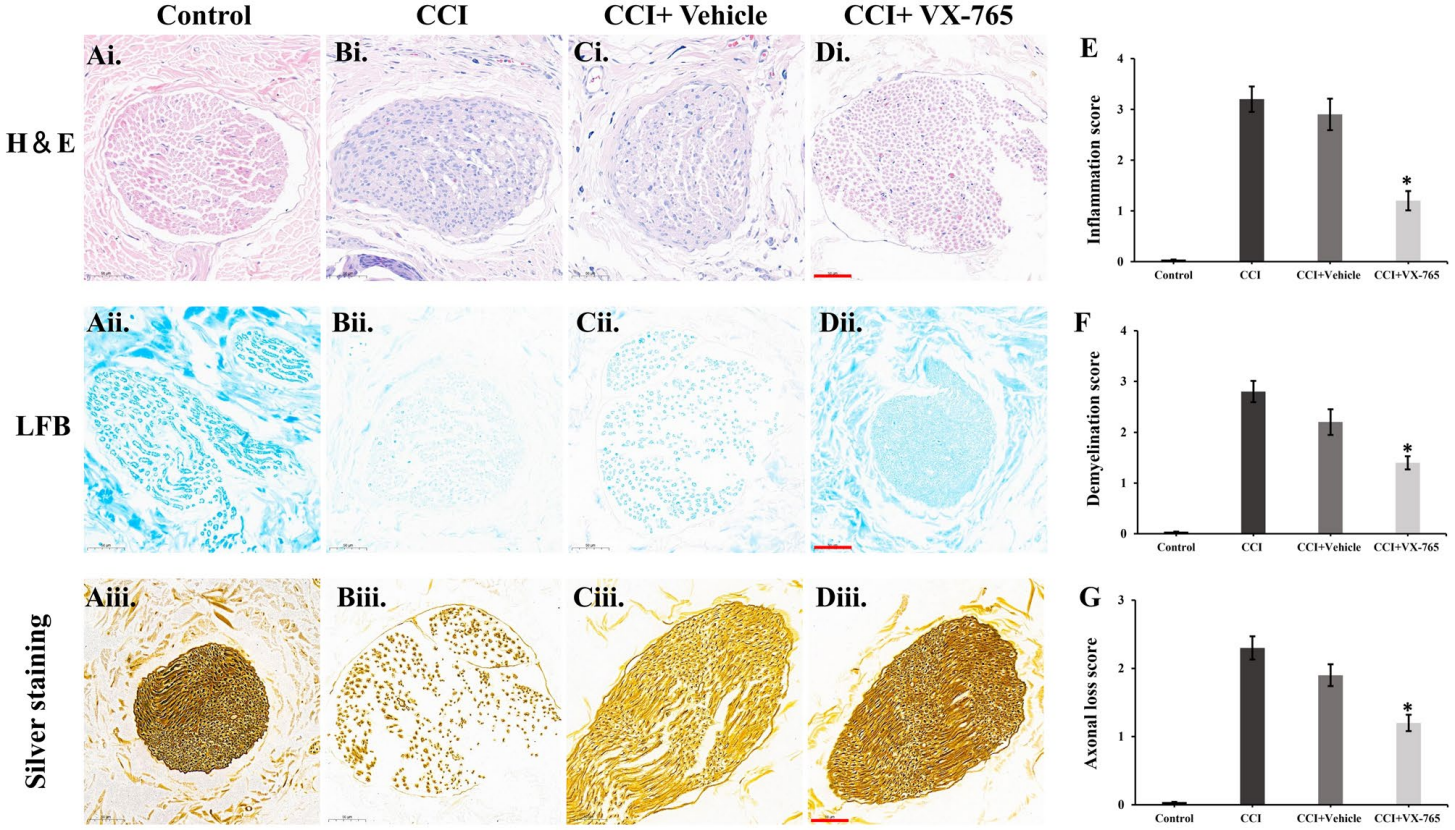
The histopathological findings. Following treatment of VX-765, siRNAs, CoPP or Znpp, the changes of inflammatory cell infiltration are depicted in (**Ai**–**Di**, **E**). The changes in demyelination severity are shown in (**Aii**–**Dii** and **F**). The changes in axonal loss severity are displayed in (**Aiii–Diii** and **G**). Scale bar: 50 µm; *p < 0.05 vs CCI group.

We also evaluated the degree of inflammatory cell infiltration, demyelination, and axonal damage in the trigeminal nerve using various staining techniques. Hematoxylin and eosin staining revealed significant inflammatory cell infiltration in the CCI and CCI + Vehicle groups, While this infiltration decreased significantly after VX-76 treatment (Fig. 3Ai–Di). The inflammation score, assessed using a scoring system (0–4), was significant lower in the CCI + VX765 group compared to the CCI group (p < 0.05) (Fig. 3E). To assess demyelination, we used the LFB staining. The CCI group showed patchy demyelination in the trigeminal nerve, while, the CCI + VX765 group exhibited less pronounced changes, suggesting lower levels of demyelination (Fig. 3Aii–Dii). Analysis of demyelination severity scores (ranging from 0 to 3) for LFB-stained sections revealed a significant decrease in the CCI + VX765 group compared to the CCI group (Fig. 3F). Axonal damage in trigeminal nerve was assessed using Bielschowsky silver staining, which stains axons light to dark brown . The CCI group showed severe axonal loss and less pronounced staining, whereas the CCI + VX765 group exhibited less intense irregularities (Fig. 3Aiii–Diii). Our evaluation of Bielschowsky staining's axonal loss severity scores showed that the CCI + VX765 group had significantly less axonal loss than the CCI group (p < 0.05) (Fig. 3G).

### Inflammasome activation and pyroptosis in CCI model

**Figure 4:**
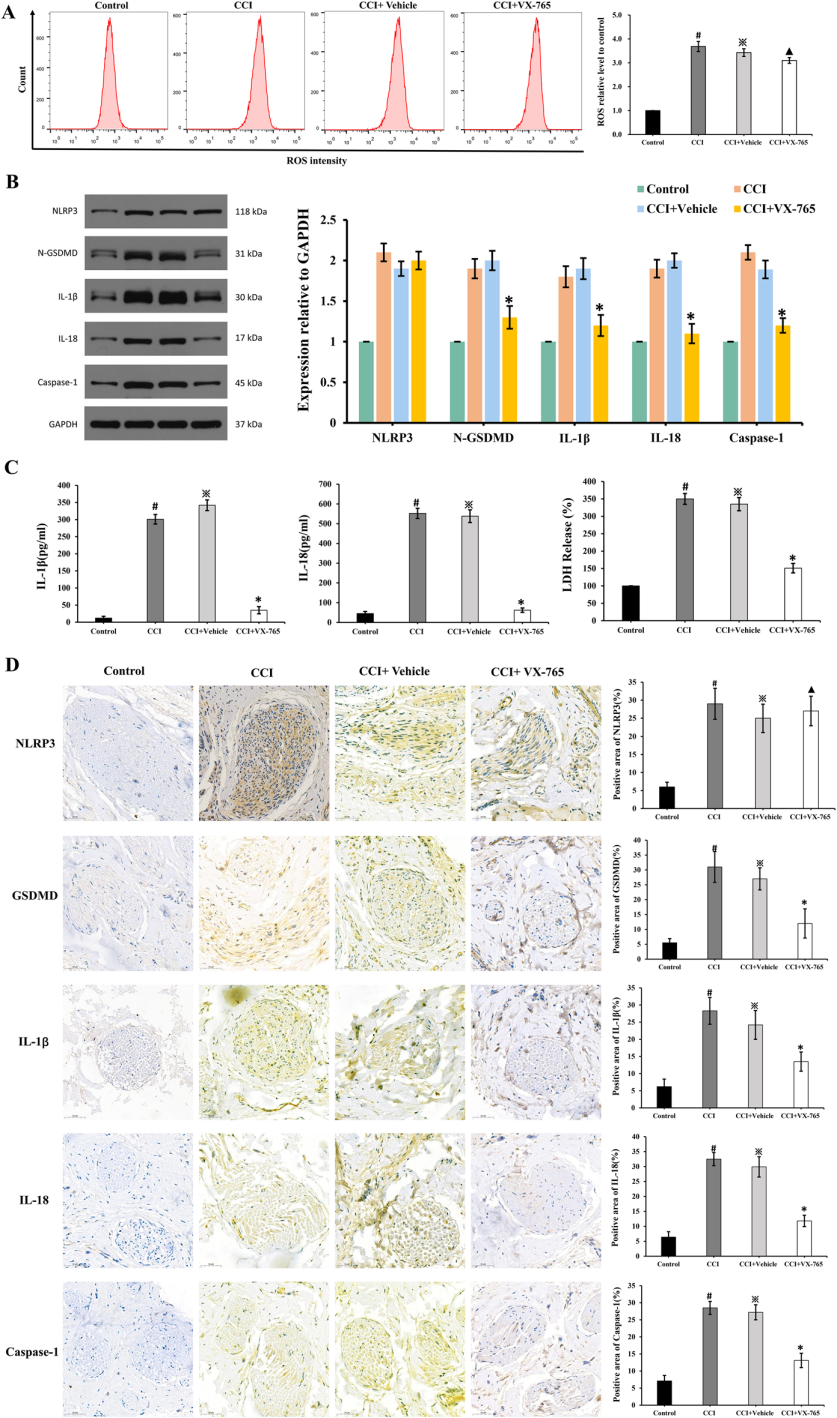
Inflammasome Activation and pyroptosis in CCI model. During the flow cytometry test, the ROS level in the CCI group was found to increase and was not affected by treatment with VX-765 (**A**). # p < 0.05 vs control group; ※p > 0.05 vs CCI group; ▲p < 0.05 vs CCI group. According to Western blot analysis, the expression of pyroptosis-related proteins increased in the CCI group, (**B**). *p < 0.05 vs CCI group. The levels of IL-1, IL-18 and LDH were significantly higher in the CCI group, but significantly lower after VX-765 treatment (**C**). # p < 0.05 vs control group; ※p > 0.05 vs CCI group; *p < 0.05 vs CCI group. (**D**) Demonstrates that the CCI model significantly increased the expression of NLRP3, caspase-1, IL-1, IL-18, and GSDMD. #p < 0.05 vs control group; ※p > 0.05 vs CCI group; ▲p < 0.05 vs CCI group. Scale bar: 50 µm.

The level of ROS in the CCI group increased compared to the control group during the flow cytometry test. However, treatment with VX-765 did not impact the level of ROS. (Fig. 4A). The expression of pyroptosis-related proteins NLRP3, GSDMD, IL-1, IL-18, and caspase-1 increased in the CCI group, according to Western blot analysis. However, after VX-765 treatment, the expression of GSDMD, IL-1, IL-18, and caspase-1 decreased (Fig. 4B). The levels of IL-1 and IL-18, measured by ELISA, were significantly higher in the CCI group but significantly lower after treatment with VX-765 (Fig. 4C). The LDH Release Assay revealed a similar pattern of LDH release as that of IL-1 and IL-18. Figure 3D demonstrates that the CCI model significantly increased the expression of NLRP3, caspase-1, IL-1, IL-18, and GSDMD. Except for NLRP3, the expression of caspase-1, IL-1, IL-18, and GSDMD was significantly reduced after VX-765 treatment (Fig. 4D).

### H_2_O_2_ induces pyroptosis in RSC96 Cells

**Figure 5:**
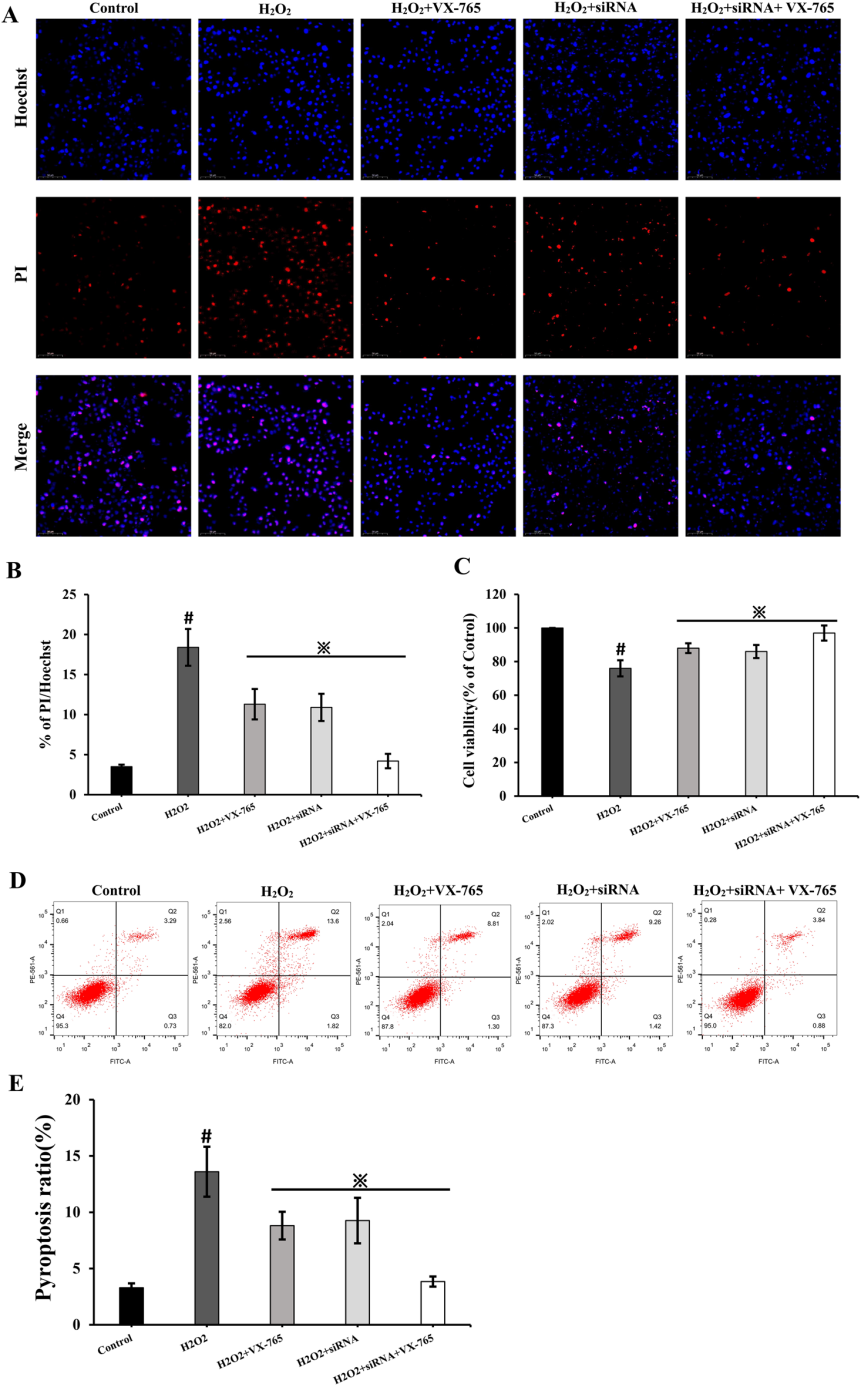
Demyelination of TN nerve and neurobehavioral changes of CCI model. The changes of pyroptosis percentage and relative cell viability showed in (Fig. **A**, **B**, **C**). # p < 0.05 vs control group; ※p < 0.05 vs H_2_O_2_ group. Scale bar: 50 µm. The percentage of pyroptosis significantly increased in the H_2_O_2_ group and reduced significantly in the H_2_O_2_ + VX-765 group, the H_2_O_2_ + siRNA group, and the H_2_O_2_ + VX-765 + siRNA group (Fig. **D**, **E**). # p < 0.05 vs control group; ※p < 0.05 vs H_2_O_2_ group.

To identify the type of cell death induced by H_2_O_2_, RSC96 cells were stained with Hoechst33324/PI and analyzed under a fluorescence microscope. The percentage of cells positive for PI/Hoechst staining significantly increased in the H_2_O_2_ group (Fig. 5A, B) However, when cells were treated with VX-765 and siRNA, the percentage of PI/ Hoechst -positive cells markedly decreased in the H_2_O_2_ + VX-765 group, H_2_O_2_ + siRNA group, and H_2_O_2_ + VX-765 + siRNA group (Fig. 5A, B). According to the CCK-8 results, relative cell viability decreased significantly in the H_2_O_2_ group. In contrast, after treatment with VX-765 and siRNA, relative cell viability increased significantly when compared to the H_2_O_2_ group (Fig. 5C). Furthermore, after treating RSC96 cells with H_2_O_2_, we measured the percentage of cell viability and pyroptosis using annexin V-FITC/PI and flow cytometry. Consistent with the previous results, H_2_O_2_ treatment significantly increased the rate of pyroptosis and and decreased the cell viability of RSC96 cells (Fig. 5D, E). In contrast, treatment with VX-765 and siRNA significantly reduced the rate of pyroptosis significantly in the H_2_O_2_ + VX-765 group, the H_2_O_2_ + siRNA group, and the H_2_O_2_ + VX-765 + siRNA group (Fig. 5D, E). According to the aforementioned results, H_2_O_2_ strongly promoted pyroptosis in RSC96 cells.

### Inflammasome activation and pyroptosis in RSC96 Cells

**Figure 6:**
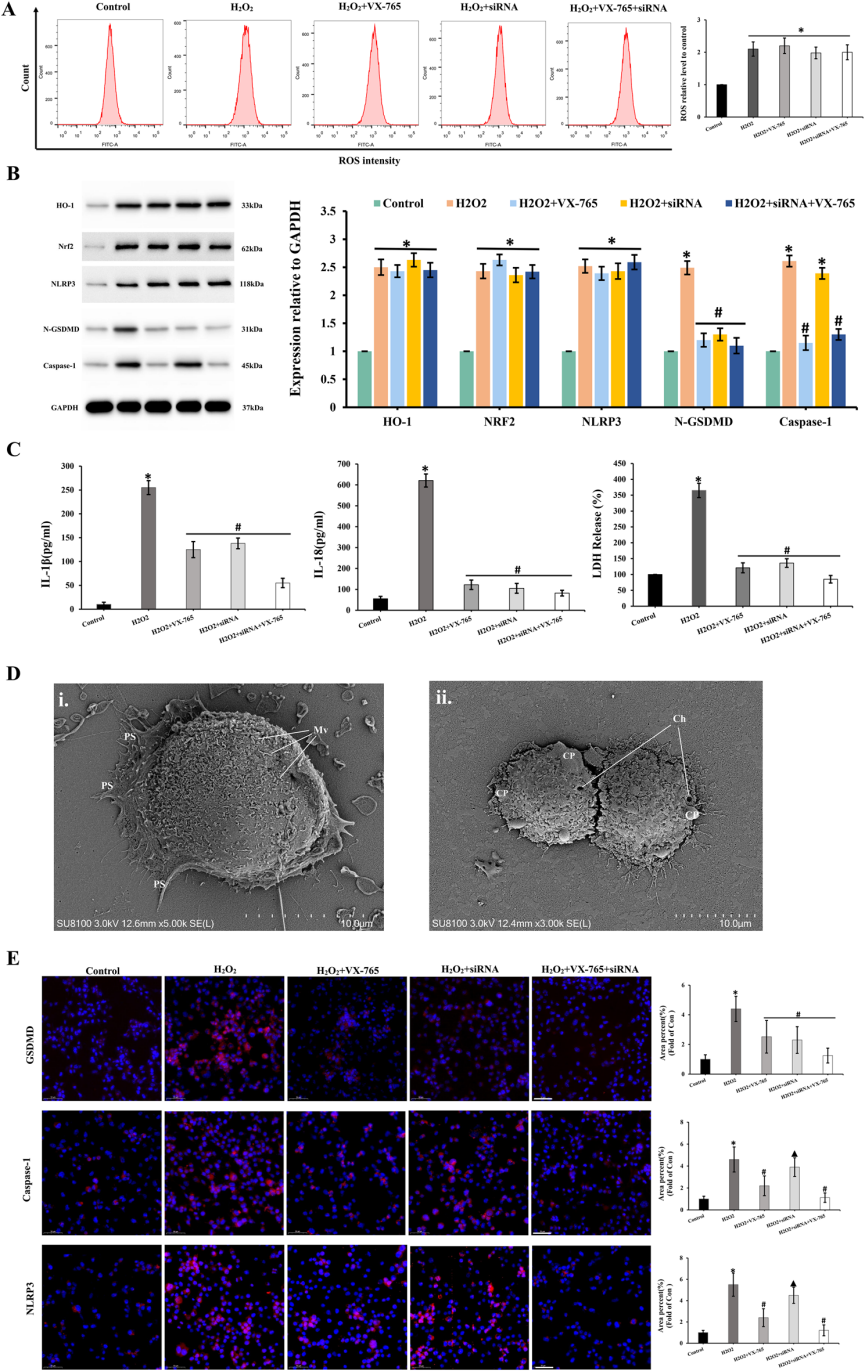
Inflammasome Activation and pyroptosis in RSC96 Cells. The ROS level in the H_2_O_2_ group increased during the flow cytometry test and was not affected by treatment with VX-765 (**A**). *p < 0.05 vs control group. According to Western blot analysis, the expression of pyroptosis-related proteins increased in the H_2_O_2_ group, (**B**). *p < 0.05 vs control group; # p < 0.05 vs H_2_O_2_ group. The levels of IL-1, IL-18 and LDH were significantly higher in the H_2_O_2_ group and significantly lower after VX-765 or siRNA treatment (**C**). *p < 0.05 vs control group; # p < 0.05 vs H_2_O_2_ group. The RSC96 cells exhibitedmild pyroptosis and overall mild edema after H_2_O_2_ treatment (**D**). Scale bar: 10 µm. CP: Cell Process; Ch: Cell hole; Ps: Pseudopodium; Mv: Microvilli. Figure 6E showed that the expression of caspase-1, NLRP3, and GSDMD was significantly increased in the H_2_O_2_ group. *p < 0.05 vs control group; # p < 0.05 vs H_2_O_2_ group. Scale bar: 50 µm.

The ROS level in the H_2_O_2_ group increased compared with control group, and treatment with VX-765 and siRNA had no effect on the level of ROS (Fig. 6A). The expression of pyroptosis-related proteins NLRP3, GSDMD, and caspase-1 increased in the H_2_O_2_ group, according to Western blot analysis. NLRP3 expression did not change significantly after VX-765 or siRNA treatment (Fig. 6B). When compared to the H_2_O_2_ group, the expression of GSDMD decreased significantly after treatment with VX-765 or siRNA (Fig. 6B). Caspase-1 expression did not change after treatment of siRNA but decreased significantly after treatment of VX-765 (Fig. 6B). After being exposed to H_2_O_2_, Nrf2 and its effector molecule HO-1 were found up-regulated significantly at the transcriptional level (Fig. 6B). In addition, the treatment of VX-765 or siRNA did not change the level of Nrf2 and HO-1. The ELISA results showed that the levels of IL-1 and IL-18 were significantly higher in the H_2_O_2_ group and significantly lower after VX-765 or siRNA treatment (Fig. 6C). The LDH Release Assay demonstrated that the LDH levels altered in a manner that paralleled the changes observed in IL-1 and IL-18. The scanning electron microscope was also used to detect pyroptosis in RSC96 cells caused by H_2_O_2_. The overall structure of the RSC96 cells in the control group was integrity. The cell membrane was intact, and there were numerous pseudopodia around the cells. Microvilli were plentiful without obvious swelling, and extracellular matrix was abundant (Fig. 6Di). The cell membrane structure was blurred and disintegrated, there were a few fenestrations on the surface, and the cell pseudopodia were broken and degraded (Fig. 6Dii). Figure 5E showed that the expression of caspase-1, NLRP3, and GSDMD was significantly increased in the H_2_O_2_ group. The expression of NLRP3, caspase-1, and GSDMD was significantly decreased after treatment of VX-765 (Fig. 6E). The expression of GSDMD decreased significantly after treatment of siRNA, whereas NLRP3, caspase-1 expression was unaffected (Fig. 6E).

### The Nrf2/HO-1 signaling pathway plays a role in pyroptosis

**Figure 7:**
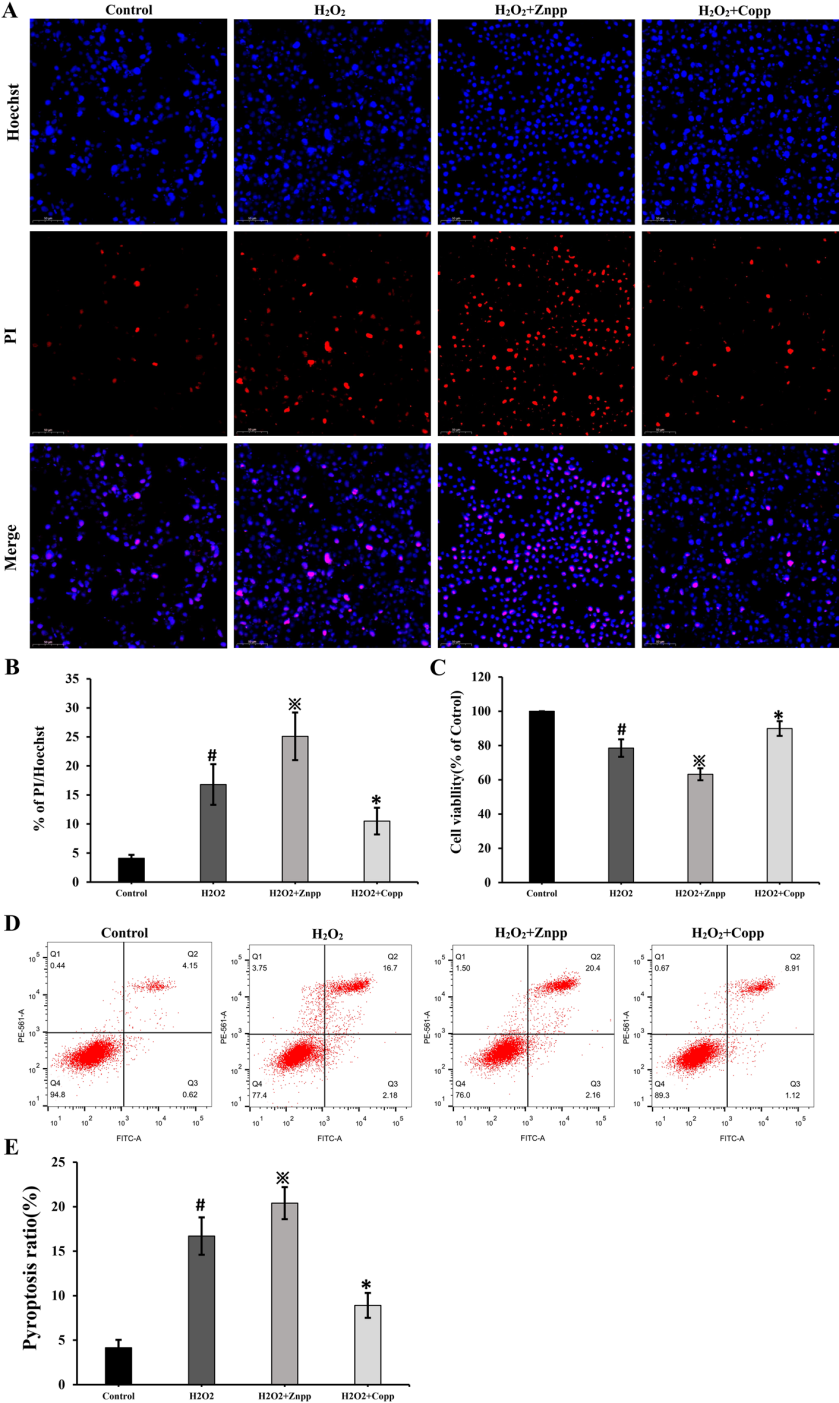
The changes of pyroptosis in RSC96 Cells after treatment of Znpp or Copp. As shown in (**A, B**), after treatment of Znpp or Copp, the percentage of pyroptosis increased or decreased significantly. # p < 0.05 vs control group; ※p < 0.05 vs H_2_O_2_ group; *p < 0.05 vs H_2_O_2_ group. Scale bar: 50 µm. The CCK-8 result showed that relative cell viability changed correspondingly (**C**). # p < 0.05 vs control group; ※p < 0.05 vs H_2_O_2_ group; *p < 0.05 vs H_2_O_2_ group. The flow cytometry data revealed that H_2_O_2_ enhanced the rate of pyroptosis while decreasing the cell viability of RSC96 cells (**D, E**). # p < 0.05 vs control group; ※p < 0.05 vs H_2_O_2_ group; *p < 0.05 vs H_2_O_2_ group.

As depicted in Fig. 7A, B the percentage of PI/ Hoechst increased significantly in H_2_O_2_ group. While, after treatment of Znpp, the percentage of PI/ Hoechst increased significantly compared to the H_2_O_2_ group, which indicated that the pyroptosis increased (Fig. 7A, B). After treatment with Copp, the percentage of PI/ Hoechst decreased significantly compared to H_2_O_2_ group, indicating a decrease in pyroptosis (Fig. 7A, B). The CCK-8 result revealed that relative cell viability decreased significantly in the H_2_O_2_ group. While, after treatment of Znpp, the relative cell viability decreased significantly compared with the H_2_O_2_ group (Fig. 7C). After treatment of Copp, the relative cell viability increased significantly compared with the H_2_O_2_ group (Fig. 7C). The flow cytometry data demonstrated that H_2_O_2_ enhanced the rate of pyroptosis (Fig. 7D, E). Following treatment with Znpp, the rate of pyroptosis significantly increased compared with the H_2_O_2_ group (Fig. 7D, E). After treatment of Copp, the rate of pyroptosis decreased significantly compared with the H_2_O_2_ group (Fig. 7D, E). Taken together, the above results suggested that Nrf2/HO-1 signaling pathway plays a role in pyroptosis.

**Figure 8:**
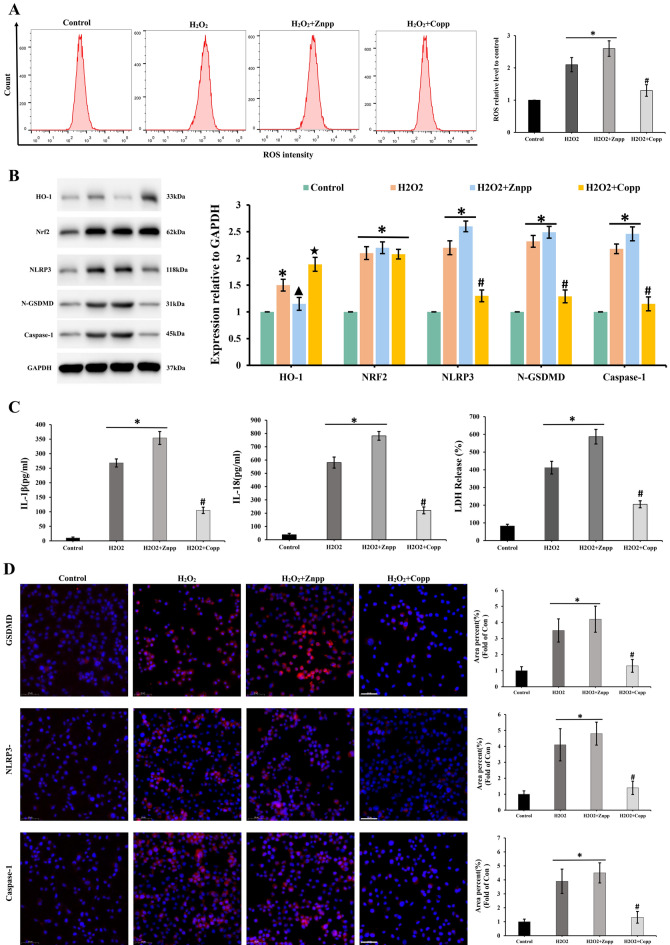
The role of Nrf2/HO-1 signaling pathway in pyroptosis. During flow cytometry test, the ROS level in the H_2_O_2_ group increased, and the treatment with Znpp and Copp did not influence the level of ROS (**A**). *p < 0.05 vs control group; #p < 0.05 vs H_2_O_2_ group. The expression of pyroptosis-related proteins NLRP3, GSDMD, caspase-1, Nrf2 and HO-1 increased in the H_2_O_2_ group, as shown by Western blot results (**B**). *p < 0.05 vs control group; #p < 0.05 vs H_2_O_2_ group; ▲p < 0.05 vs H_2_O_2_ group; ★p < 0.05 vs H_2_O_2_ group. The levels of IL-1, IL-18 and LDH increased considerably in the H_2_O_2_ and H_2_O_2_ + Znpp groups and dropped considerably following Copp therapy (**C**). *p < 0.05 vs control group; #p < 0.05 vs H_2_O_2_ group. According to the immunofluorescence results in (**D**), the expression of NLRP3, caspase-1, and GSDMD greatly elevated in both the H_2_O_2_ and H_2_O_2_ + Znpp groups and significantly decreased after treatment of Copp. *p < 0.05 vs control group; #p < 0.05 vs H_2_O_2_ group. Scale bar: 50 µm.

Compared with the control group, the ROS level in the H_2_O_2_ group increased during flow cytometry test and the treatment with Znpp and Copp did not influence the level of ROS (Fig. 8A). According to Western blot results, there was an increased expression of pyroptosis-related proteins, namely NLRP3, GSDMD, caspase-1, Nrf2 and HO-1 in the H_2_O_2_ group. After treatment with Znpp, the expression of NLRP3, GSDMD, caspase-1 and Nrf2 did not change significantly compared with the H_2_O_2_ group. While, the level of HO-1 decreased significantly. Conversely, Copp treatment led to a significant decrease in the expression of NLRP3, GSDMD, caspase-1 compared to the H_2_O_2_ group. While, the level of Nrf2 did not change significantly (Fig. 8B). The ELISA results revealed that the levels of IL-1 and IL-18 increased considerably in the H_2_O_2_ and H_2_O_2_ + Znpp groups. When comparison to the H_2_O_2_ group, the levels of IL-1 and IL-18 dropped considerably following Copp therapy (Fig. 8C). The LDH Release Assay showed the level of LDH changed similar with IL-1β and IL-18. According to the immunofluorescence results in Fig. 7D, both the H_2_O_2_ and H_2_O_2_ + Znpp groups greatly elevated the expression of NLRP3, caspase-1, and GSDMD. After treatment of Copp, the expression of NLRP3, caspase-1, and GSDMD significantly decreased compared with H_2_O_2_ group (Fig. 8D).

## Discussion

In our study, we confirmed that trigeminal nerve demyelination occurred in the CCI model. The SEM revealed the presence of a ball-like bulge and membrane pore formation in the TN nerve of the CCI models, indicating the generation of pyroptosis in the myelin sheath. Pathology detection discovered increased inflammatory cell infiltration, demyelination, and axonal loss in the CCI model. The treatment with VX765 improved the histopathological changes in the CCI model. We also observed an increase in ROS level, as well as pyroptosis related proteins such as NLRP3, GSDMD, IL-1, IL-18, and caspase-1, and LDH levels in the CCI model, while the expression of these factors decreased after treatment with VX-765. The findings further confirmed the occurrence of pyroptosis in the TN nerve of the CCI model. To study the mechanism of pyroptosis in vitro, we used H_2_O_2_-treated RSC96 cells. Hoechst/PI and flow cytometry tests showed an increased in H_2_O_2_-induced lytic cell death. In the H_2_O_2_-treated RSC96, the SEM revealed that the cell membrane structure was blurred and disintegrated, a few fenestrations appeared on the surface, and the cell pseudopodia were broken and degraded. We also observed an increase in ROS level, as well as pyroptosis related proteins and LDH level in H_2_O_2_-treated RSC96. However, the expression of the above factors decreased after treatment with VX-765 or siRNA. Treatment with Znpp or Copp altered the rate of pyroptosis and pyroptosis-related proteins in H_2_O_2_-treated RSC96 cells, suggesting the involvement of Nrf2/HO-1 signaling pathway in pyroptosis.

While it is widely accepted that the primary cause of TN pain is mechanical stimulation of the trigeminal nerve caused by impression of blood vessel^2^. However, mechanical stimulation alone cannot explain TN pain. As a result, additional underlying processes have been sought by researchers^17,18^. One of the most noticeable processes is the demyelination of the nerve at the point of compression. Burchiel et al.^6^ discovered that after inducing demyelination of cat trigeminal nerve roots, the animal exhibited action potentials in response to stimulation. The preclinical study above demonstrated the link between demyelination and TN-like changes. Hilton and colleagues revealed demyelination at the site of arterial compression using electron microscopy, which firstly reported the clinical evidence of demyelination associated with TN^5^. Rappaport et al.^19^ verified this discovery by analyzing biopsy samples collected from the site of compression in 12 patients with TN following microvascular decompression. In our study, we also confirmed the occurrence of demyelination in the trigeminal nerve using electron microscopy in the CCI model. Additionally, scanning electron microscopy (SEM) identified pyroptosis in the CCI model and H2O2-induced RSC96 cells. These findings suggest that pyroptosis may contribute to demyelination.

Pyroptosis, a type of proinflammatory programmed cell death mediated by GSDMD, can also be triggered by proinflammatory caspase activation. GSDMD connects to inner membrane lipids and oligomerizes, forming membrane holes and lead to localized cellular swelling, membrane rupture, and cellular content extravasation^20,21^. Activation of inflammasomes and pyroptosis have been documented in a range of CNS cell types. Human microglia, neurons, myelin-forming oligodendrocytes (ODCs), and astrocytes all exhibit robust NRLP3-associated inflammasome responses^22,23^. Several CNS illnesses, including Alzheimer's, traumatic brain/spinal cord damage, and epilepsy, have been associated to the formation of inflammasomes^24,25^. IL-1 and IL-18, crucial elements of the NLRP3 inflammasome signaling pathway, are derived from their pro-IL-1 and pro-IL-18 predecessors, which may be activated by functioning caspase-1, respectively. In the experimental autoimmune encephalomyelitis model, IL-1 has neurotoxic effects by increasing BBB permeability and hastening leukocyte infiltration^26^. Despite the speculation about pyroptosis in these diseases, the role of GSDMD in cranial nerve demyelination remains unexplored. This study hypothesizes that NLRP3 inflammasome and GSDMD-related pyroptosis could regulate inflammatory responses and demyelination. Our research demonstrated that the levels of ROS, pyroptosis-related proteins (including NLRP3, GSDMD, IL-1β, IL-18, and caspase-1), and LDH increased in the CCI model and H2O2-treated RSC96. After VX-765 treatment, the expression of these factors decreased. These findings confirm the occurrence of pyroptosis in the TN nerve of the CCI model and H2O2-treated RSC96.

According to emerging evidence, various endogenous elements such as adenosine triphosphate and uric acid, as well as exogenous substances such as silica, asbestos, and alum, activate the NLRP3 inflammasome^27,28^. It has recently been discovered that these activators lead to an increased cellular production of reactive oxygen species (ROS), which occurs prior to the activation of the NLRP3 inflammasome^29,30^. HO, as a rate-limiting enzyme that catalyzes heme metabolism, is essential for the control of oxidative stress and mitochondrial function. Oxidative stress, infection, inflammation, and ischemia are all known to induce the expression of HO-1. Furthermore, HO-1 participates in a wide range of stress reactions, including physical, chemical, and biological stressors^31–33^. Studies have demonstrated that the Nrf2 pathway can regulate HO-1 expression at the heme level^34,35^. Previous research found that ROS caused oxidative stress and, as a result, activated HO-1. In our study, we discovered that after treatment with the HO-1 inhibitor Znpp or its activator Copp, the pyroptosis-related proteins changed in the H2O2-treated RSC96. The above findings suggested that the Nrf2/HO-1 signaling pathway is involved in pyroptosis^36^. The HO-1/Nrf2 pathway is strongly linked to oxidative stress. Our study measured oxidative stress levels in rats and found that HO-1 can efficiently regulate oxidative stress levels in organisms. The ERK signaling pathway may be stimulated to promote mitochondrial production, which is lowered once the HO-1/ Nrf2 pathway is suppressed, balancing external stimulus^37,38^. However, one limitation of this study is that the epigenetic mechanism by which ROS regulates Nrf2/HO-1 in real-life situations remains unclear. Therefore, further investigation is needed to understand the specific interaction between ROS and Nrf2/HO-1 in real-life scenarios.

## Conclusions

**Figure 9:**
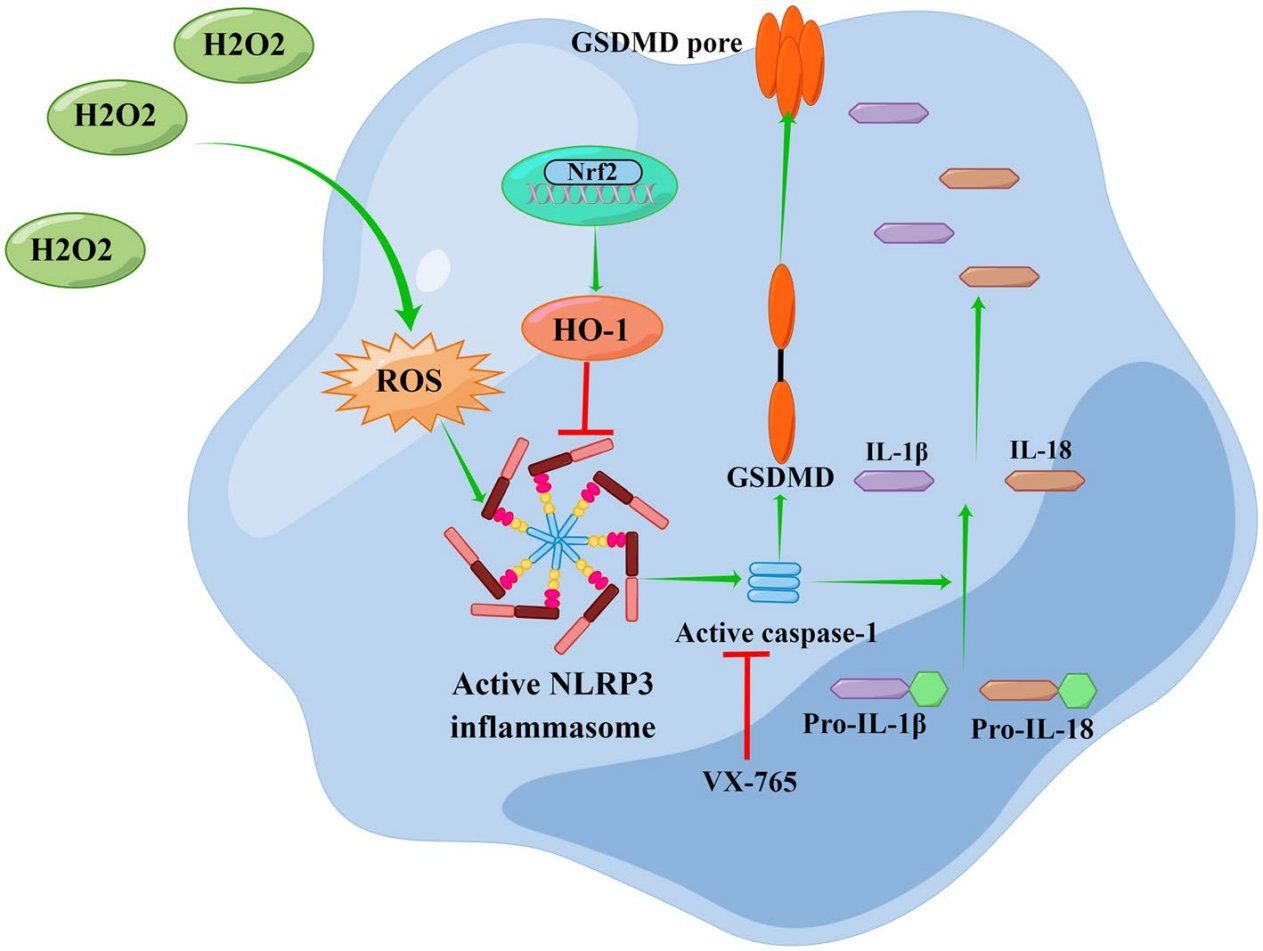
The mechanism of pyroptosis in CCI model. The ROS is an upstream event of NLRP3 inflammasome activation which induced eventual pyroptosis. The Nrf2/HO-1 signaling pathway could protect the H2O2-induced pyroptosis in RSC96 cells.

In conclusion, the pyroptosis of Schwann cell in the CCI model generated the demyelination of TN nerve. The ROS is an upstream event of NLRP3 inflammasome activation which induced eventual pyroptosis. The Nrf2/HO-1 signaling pathway could protect the H_2_O_2_-induced pyroptosis in RSC96 cells. The possible mechanism of pyroptosis in CCI model was shown in Fig. 9. In the real life, the ROS may induce pyroptosis of Schwann cell, which would result in demyelination of TN nerve. However, the mechanism of ROS involved in the regulation of Nrf2/HO-1 in real life remains unclear. This study provides a new idea for studying the demyelination of TN nerve and a new strategy for the treatment of TN. Removal of H_2_O_2_-induced pyroptosis may provide a favorable microenvironment for axonal regeneration, which is beneficial for nerve regeneration and functional recovery. This therapy may solve the problem of demyelination of TN nerve and provides a new method for the treatment of TN.

### Supplementary Information

Supplementary Information.

## Data Availability

Researchers with reasonable requests to access deidentified summary data and analysis script should contact the corresponding author.

## Abbreviations

TN: Trigeminal neuralgia
CCI: Chronic constriction injury of the infraorbital nerve
HE: Hematoxylin and eosin
ROS: Reactive oxygen species
MS: Multiple sclerosis
IL-1: Interleukin-1
IL-18: Interleukin-18
GSDMD: Gasdermin D
