# Supplementary material for: Attenuates reactive oxygen species: induced pyroptosis via activation of the Nrf2/HO-1 signal pathway in models of trigeminal neuralgia
Source: Sci Rep. 2023 Oct 23;13:18111. doi: 10.1038/s41598-023-44013-w (PMC10593956; doi:10.1038/s41598-023-44013-w)
Supplement: Supplementary file 1 — Supplementary Information. [file 41598_2023_44013_MOESM1_ESM.docx]

**METHODS**

***Reagents, Antibodies and Plasmids***

Belnacasan (VX-765, T6090) was purchased from Topscience Co., Ltd. (Shanghai, China). Hoechst 33342 (B2261) and propidium iodide (PI, P4170) were bought from Sigma (St. Louis, MO, USA). Annexin V-FITC/PI Apoptotic detection kit was purchased from Vazyme Biotech Co., Ltd. (Nanjing, China). The primary antibodies used in the current study were as follows: IL-1β (12242) and NLRP3 (15101) from Cell Signaling Technology Inc, (CST, Beverly, MA, USA); Caspase-1 (A1115) and IL-18 (A0964) from ABclonal Biotechnology Co., Ltd. (Wuhan, China); β-actin (M177-3) from MBL International (Woburn, MA, USA); GSDMD from Proteintech Group, Inc., (Wuhan, China). Human IL-1β ELISA Kit (CHE0001) and human IL-18 ELISA Kit (CHE0007) were purchased from 4A Biotech Co., Ltd., (Beijing, China). ZnPP (purity ≥ 92%) were purchased from Sigma (SigmaAldrich, USA); H_2_O_2_ and cobalt protoporphyrin (CoPP) were purchased from J&K Scientific (Beijing, China).

**Animals**

In our study, we used the adult male Sprague-Dawley rats with weighing 180-220 g. The experimental procedures were approved by the Institutional Animal Care and Use Committee of Qingdao University. The animals were raising with water and food *ad libitum* in standard laboratory conditions. Efforts were made to minimize the animals’ number and discomfort.

**Surgery and Drug Administration**

The chronic constriction injury of infraorbital nerve (CCI) was used to establish the trigeminal neuropathic pain model. The operation was proceeded under anesthesia (pentobarbital, 50 mg/kg, intraperitoneally) after 14 days of presurgical adaptation training. Without damaging the whiskers, the region between the left eye and whisker pad was shaved. A 3-cm long incision from the third row of whisker lines was made caudally to the orbit. After separating the superficial fascia, the infraorbital nerve was exposed and two 4-0 chromic catgut ligatures with a 3-mm length of interval were used to tie the nerve loosely to reduce the nerve diameter noticeably and retard the superficial vasculature but not to cut off. Finally, the skin incision was closed with a polyester suture. The animals in sham groups underwent the same surgical procedure except for the actual nerve ligation. According to previous studies, we regarded the successful trigeminal neuropathic pain model as pain threshold was lower than 2 g.

To analyze the effect of VX-765 on neurobehavioral and neuropathological outcomes, CCI animals received daily i.p. injections of VX-765 (50 mg/kg) diluted in PBS/DMSO from the onset of clinical signs (day 14) until the end of the experiment. Vehicle treated animals received daily injections of PBS/DMSO.

The neurobehavioral including mechanical threshold, dynamic allodynia and pinprick hyperalgesia were detected for the CCI models with or without administration of VX-765. The electron microscopy was used to analyzed the demyelination of trigeminal nerve. The HE staining, LFB staining and Bielschowsky’s method were used to detect the histochemical changes of trigeminal nerve. The immunohistochemistry, Elisa and western blotting were used to detect the expression of GSDMD, caspase-1, NLRP3, IL-18 and IL-1β. The flow cytometry was used to measure the levels of ROS.

**Cell Culture and Drug Treatment**

RSC96 cells (rat Schwann cell line, BCRC No. 60507) were purchased from Procell Life Science & Technology Co.,Ltd (Wuhan, China). RSC96 cells were maintained in Dulbecco’s modified Eagle’s medium (DMEM) (Thermo Fisher Scientific, Waltham, MA, USA) containing 5.6-mM glucose, 4-mM L-glutamine plus with 100 U/mL penicillin, 100-µg/mL streptomycin and 10% (v/v) fetal bovine serum (Thermo Fisher Scientific) at 37 ℃ in 5% CO_2_ humidified atmosphere. The medium was changed every 2–3 days. At 70% confluence cells were synchronized by serum starvation for 4 hr. All experiments were performed within 10 cell passages.

All cell groups were treated with H_2_O_2_ at 80% confluence (300 µM). RSC96 cells were exposed to VX-765 (50 μM, 24 h pretreatment, catalog no. inh-vx765-1; InvivoGen), or a mixture of three commercially available GSDMD-targeting Dicer-Substrate siRNAs (30 nM), or solvent control for 24 h. Cells in the group receiving CoPP or Znpp treatment were pretreated with CoPP (40 mM) or ZnPP (10 μM) for 24 h and then incubated with H_2_O_2_ for an additional 24 h. Supernatants were harvested and stored at −80 °C.

**siRNA Knockdown of GSDMD**

Cells were transfected with 30 nM of nontargeting siRNA (TriFECTa RNAi Kit; Integrated DNA Technologies) or a mixture of three commercially available GSDMD-targeting Dicer-Substrate siRNAs (30 nM), in combination with PrecisionFectin Transfection Reagent (BioIntersect). After 48 h, cells were exposed to H_2_O_2_, or solvent control (with or without VX-765) for 24 h.

**Cell Viability Assays**

Cell viability was detected with a Cell Counting Kit-8 (CCK-8, Biotools, Taipei, Taiwan). After different treatment, the medium was refreshed and 10 µL of the CCK-8 solution was added to each well. After incubation for 2 h at 37 ◦C at 5% CO2, optical absorbance at 450 nm (with 650 nm as reference) was measured using a microplate reader (Synergy™ H1, BioTek, Winooski, VT, USA). An average was calculated from three independent experiments. Cell viability was calculated as [(Treated: A450–A650) − (Blank: A450–A650)]/ [(control: A450–A650) − (Blank: A450–A650)] × 100%.

**Cell Death Assay**

Cell death was assessed by staining with Hoechst 33342 (B-2261, Sigma-Aldrich, St. Louis, MO, USA) and propidium iodide (PI, P4170, Sigma-Aldrich) followed by fluorescence microscopy (Zeiss LSM710; Carl Zeiss, Oberkochen, Germany). Briefly, RSC96 cells were plated at an initial density of 1 × 104 cells/well in 24-well plates. After different treatments, the nuclei were stained by Hoechst 33342 (10 µM) for 15 min at room temperature, and then dead cells were stained with PI (5 µg/mL) for 15 min in the dark. The cells were observed immediately by Zeiss LSM710 microscope, capturing six random fields for each group. Cell death was quantitated as the percentage of PI-positive cells relative to the total cell number (Hoechst 33342-positive cells). All experiments were performed at least three times.

**Flow Cytometry Analysis**

In our study, pyroptosis was measured by the annexin V-FITC/PI Detection Kit from BD Biosciences (San Jose, CA, United States), and the pyroptotic (PI positive) and apoptotic (Annexin V-FITC positive) cells could be successfully analyzed. After different treatment, cells were harvested, washed with PBS twice and stained using the Annexin V-FITC/PI Apoptosis assay kit by following the manufacturer’s instructions. After incubation at room temperature for 15 min in the dark, the stained cells were analyzed on the FACSVerse flow cytometer (BD Biosciences, San Jose, CA, USA). Data acquisition and analysis were performed using the Flowjo software (BD Biosciences, San Jose, CA, USA).

**Innocuous touch assays**

*Mechanical threshold*

The investigator blinded to the treatment conditions performed the behavioral experiment. With 20 von Frey filaments (North Coast, USA), the mechanical pain threshold was detected. Before the test, the plastic cage environment was used to adapt the rat for at least 1 hour. We stimulated the nerve-injured side whisker pad three times with a 30-seconds interval for six times. Each stimulation began with the lowest force filament and the test was repeated with increasing stiffness filaments until a filament induced one of the following behaviors twice: escape or attack reactions, brisk head withdrawal or short-lasting facial grooming. According to previous studies, we regarded the successful trigeminal neuropathic pain model as pain threshold was lower than 2 g.

*Dynamic allodynia*

The animals were acclimated in von Frey chambers for 1 hour. The 5/0 brush was used to stroke the center of the whisker pad gently. The response was scored as follows: 0 for no response; 1 for very short, fast movement of the head; 2 for sustained withdrawal of the head for more than two seconds; and 3 for flinching, licking, or flicking of the whisker pad. The allodynia score was reported as the average scores across three trials per rats.

**Pain assays**

*Pinprick hyperalgesia*

After the chronic contraction injury, the response to pinprick was scored as follows: 0 for no movement, 1 for short withdrawal, 2 for sustained withdrawal, and 3 for flinching or licking of affected whisker pad. The hyperalgesia score was reported as the average score across five trials per mouse.

**Specimens**

For specimens preparing, with pentobarbital anesthesia (50mg/kg, intraperitoneally), the animals were perfused through the ascending aorta with saline followed by 4% paraformaldehyde in 0.1 M phosphate buffer (4℃, pH 7.2–7.4). We removed the ipsilateral TG after the skull was opened and stored the TG in liquid nitrogen.

**Electron microscopy**
At Day 14, the animals were killed with cervical dislocation and a 5-mm long trigeminal nerve sample around the ligated point were obtained for the next experiments. The trigeminal nerve specimen was fixed in 4% paraformaldehyde and 2% glutaraldehyde in buffer for 48 h at 4 C. After being washed 3 times with buffered solution, it was exposed to 1% osmium tetroxide for 1 h as post-fixation. Staining was achieved with 1% aqueous uranyl acetate, followed by dehydration with an ethanol gradient and treatment with propylene oxide. Sections were collected on polyvinyl Formvar–coated grids and examined using electron microscopy (H-600, Hitachi, Japan).

The RSC96 Cells were fixed with 2.5% glutaraldehyde for 3 h, and then rinsed with 0.1% PB three times. Then they were dehydrated through a graded series of ethanol (30, 50, 70, 95 and 100%) and dried by the tertiary butanol method. The samples were then mounted on metal stubs and dried in a silica gel vacuum desiccator. They were sputter coated with gold and examined under a Hitachi S-4800 scanning electron microscope (SEM) operating at 15 kV.

**Histochemical assessment**

After last behavioral observation, a 5-mm long trigeminal nerve sample around the ligated point were carefully removed for biochemical and pathological assessments. For histological evaluation, the nerve sections were stained using different staining methods to determine severity of inflammation, demyelination, and axonal loss. In this regard, the nerves were fixed in 10% formalin solution and paraffin-embedded. Next, serial sections (thickness = 8 mm) were prepared using a microtome. The sections were stained with Hematoxylin and Eosin to judge inflammatory infiltration, Luxol fast blue (LFB) to analyze demyelinated intensity, and Bielschowsky’s method to study axonal damage and loss. Severity of inflammation was determined in 5 sections from each animal in a blinded manner. Level of inflammation was scored as follows: 0, no inflammation; 1, cellular infiltration around blood vessels and meninges; 2, mild parenchymal infiltration; 3, moderate parenchyma infiltration; and 4, severe parenchymal infiltration. In addition, scoring for demyelination in LFB-stained sections was as follows: 1, normal myelination, 2, mild demyelination, and 3, moderate to severe demyelination. In addition, the following scoring system was used for axonal damage and loss: 0, no loss; 1, few foci of axonal loss; 2, foci of deep axonal loss; and 3, diffuse and massive axonal loss. For immunohistochemistry, sections were stained using antibodies targeting NLRP3, GSDMD, IL-1β, IL-18 or caspase-1followed by incubation with secondary antibody (HRP-conjugated, 1:100 dilution, Santa Cruz Biotechnology, Inc., Texas, USA). Binding was visualized using 3,3´-diaminobenzidinetetrahydrochloride and mild counterstaining with Hematoxylin. The positive areas per mm2 were measured

**Measurement of ROS Production**

A ROS Assay Kit (Nanjing Jiancheng Bioengineering Institute, Nanjing, China) was used to measure the levels of ROS. The cells were incubated in a medium with 20μM DCFH-DA solution for 30 min at 37°C in darkness. Subsequently, the cells were trypsinized and washed with cold phosphate-buffered saline. Flow cytometry (FACSCalibur; BD Biosciences, San Jose, CA, USA) was used to measure the levels of ROS.

**Lactate Dehydrogenase (LDH) Release Assay**

The LDH release was assessed by LDH Assay Kit (Beyotime, China). The cell supernatant was harvested after treatment with indicated drugs, and LDH release was evaluated with an LDH assay kit. The absorbance at 490 nm was measured with the microplate reader.

**Western Blotting**

The total proteins were extracted from nerve tissues and RSC96 cells using a radioimmunoprecipitation assay buffer containing protease inhibitors (Beyotime, Jiangsu, China). Subsequently, a protein assay kit (Bio-Rad, Hercules, CA, USA) was used for quantification, in accordance with the protocol provided by the manufacturer. Electrophoresis on 10% sodium dodecyl sulfate-polyacrylamide gels was performed to separate equal amounts of protein (40 μg/lane), which were rapidly transferred to polyvinylidene fluoride membranes. The membranes were blocked using 5% fat-free milk for 2 h at room temperature. Proteins were incubated with the primary antibodies at the following dilutions: anti-GAPAH (1: 1,000), Nrf2 (1: 1,000), HO-1 (1: 1,000), NLRP3 (1: 1,000), IL-1β (1: 800), IL-18 (1: 800), caspase-1 (1: 2,000), and GSDMD-N (1: 1000). Purified mouse anti-rat β-actin (A5441, Sigma-Aldrich, St. Louis, MO, USA) was used to normalize the signals generated from NLRP3 (ab214185, Abcam, Cambridge, UK), Nrf2 (ab137550, Abcam), HO-1 (ab305290, Abcam),caspase-1 (A0964, ABclonal, Woburn, MA, USA), IL-1β (ab9722, Abcam), IL-18 (ab191860, Abcam), GSDMD (#93709, Cell Signaling). Tris-buffered saline and Tween 20 buffer was used to remove excessive primary antibodies. Subsequently, the membranes were incubated with an appropriate secondary antibody at 37°C for 2 h, followed by removal of excessive secondary antibody and detection of color exposure. The levels of proteins were analyzed using Image Software (NIH, USA).

**ELISA**

IL-1β and IL-18 levels in cell supernatant of RSC96 cells were detected by using a human IL-1β ELISA kit and a human IL-18 ELISA kit according to the manufacturer’s instructions. In brief, after treatment, the culture medium was collected for centrifugation at a speed of 1000× g for 10 min. The supernatants were analyzed by ELISA kit to detect the cytokines, including IL-1β and IL-18. Absorbance was measured at 450 nm by spectrophotometer (BioTek, VT Lab, USA). Standard curves for the assay system were obtained from dilutions of the standards of IL-1β and IL-18 ELISA kit; the concentrations of IL-1β and IL-18 were then obtained by extrapolation from the standard curve

**Immunofluorescence**

RSC96 cells cultured on glass coverslips were fixed with 10% neutral buffered formalin and permeabilized with 0.1% Triton X-100 in phosphate buffered saline (PBS). After blocking with 3% bovine serum albumin in PBS, the coverslips were incubated with primary antibodies to GSDMD (A10164, ABclonal, Woburn, MA, USA), caspase-1 (A0964, ABclonal, Woburn, MA, USA) and NLRP3 (ab214185, Abcam, Cambridge, UK) overnight at 4 ◦C. Next, the coverslips were incubated with Alexa Fluor 488 goat anti-rabbit IgG for 1 h at room temperature. After the primary antibody incubation, the coverslips were washed three times in 0.01 M PBS and then incubated in Cy3-conjugated donkey anti-rabbit IgG (1:300; Jackson Immuno Research, PA) or Cy3-conjugated donkey anti-goat IgG (1:300, Jackson Immuno Research, USA) for 1 hour at room temperature. All the above coverslips were treated by a mixture of FITC- and Cy3-conjugated secondary antibodies for 1 hour at room temperature. The coverslips were rinsed with 0.01 M PBS three times and mounted on a gelatin-coated slide, and air-dried. The images of the stained coverslips were captured with a fluorescence microscope attached to a CCD spot camera (LEICA DFC350FX/DMIRB, Germany) and processed with LEICA IM50 software (Germany). We measured the percentage of positive area using LEICA Qwin V3 digital image processing system (Germany). In order to identify positively stained structure, we set the density threshold above background level firstly. An average percentage of area of IR relative to the total area of the coverslips was calculated. All experiments were performed at least three times.

**Quantification and Statistics**

The data regarding the immunofluorescence and behavioral tests are presented as Mean±SD. Differences between groups were statistically analyzed using the two-way ANOVA depending on different variables with SAS 8.0 Software. The p value less than 0.05 was considered statistically significant.
